# Identification and Validation of Novel Biomarkers and Potential Targeted Drugs in Cholangiocarcinoma: Bioinformatics, Virtual Screening, and Biological Evaluation

**DOI:** 10.4014/jmb.2207.07037

**Published:** 2022-09-13

**Authors:** Jiena Wang, Weiwei Zhu, Junxue Tu, Yihui Zheng

**Affiliations:** 1 Department of Pharmacy, The Second Affiliated Hospital and Yuying Children's Hospital of Wenzhou Medical University, Wenzhou, Zhejiang 325000, P.R. China; 2 College of Pharmacy, Wenzhou Medical University, Wenzhou, Zhejiang 325035, P.R. China; 3 College of Pharmacy, Chonnam National University, Gwangju 61186, Republic of Korea

**Keywords:** Cholangiocarcinoma, miRNA, CDK1, virtual screening, molecular modeling

## Abstract

Cholangiocarcinoma (CCA) is a complex and refractor type of cancer with global prevalence. Several barriers remain in CCA diagnosis, treatment, and prognosis. Therefore, exploring more biomarkers and therapeutic drugs for CCA management is necessary. CCA gene expression data was downloaded from the TCGA and GEO databases. KEGG enrichment, GO analysis, and protein-protein interaction network were used for hub gene identification. miRNA were predicted using Targetscan and validated according to several GEO databases. The relative RNA and miRNA expression levels and prognostic information were obtained from the GEPIA. The candidate drug was screened using pharmacophore-based virtual screening and validated by molecular modeling and through several in vitro studies. 301 differentially expressed genes (DEGs) were screened out. Complement and coagulation cascades-related genes (including AHSG, F2, TTR, and KNG1), and cell cycle-related genes (including CDK1, CCNB1, and KIAA0101) were considered as the hub genes in CCA progression. AHSG, F2, TTR, and KNG1 were found to be significantly decreased and the eight predicted miRNA targeting AHSG, F2, and TTR were increased in CCA patients. CDK1, CCNB1, and KIAA0101 were found to be significantly abundant in CCA patients. In addition, Molport-003-703-800, which is a compound that is derived from pharmacophores-based virtual screening, could directly bind to CDK1 and exhibited anti-tumor activity in cholangiocarcinoma cells. AHSG, F2, TTR, and KNG1 could be novel biomarkers for CCA. Molport-003-703-800 targets CDK1 and work as potential cell cycle inhibitors, thereby having potential for consideration for new chemotherapeutics for CCA.

## Introduction

Approximately 13% of global cancer-related mortality is currently caused by hepatobiliary malignancies [1]. Cholangiocarcinoma (CCA) captures 15-20% of the primary hepatobiliary malignancies, thereby making it the second most common primary liver cancer [2]. Although CCA is relatively rare in, it is much more aggressive than other common cancers such as liver hepatocellular carcinoma, pancreatic adenocarcinoma, and breast invasive carcinoma [3]. CCA arises from epithelial cells in the bile duct [4]. Based on its anatomical location, CCA can be classified into three subtypes: intrahepatic, perihilar, and distal CCA [5]. Relatively few studies of CCA diagnosis, treatment, and prognosis have been conducted [6]. CA19-9 is the only available tumor marker for CCA, but low specificity and false-positive are regularly reported in clinical use [7]. Surgery, radiotherapy, and chemotherapy are combined for treating CCA patients, but the outcomes are limited with a small extension to survival rate[8]. At the same time, the symptoms of early-stage CCA, such as jaundice and abdominal pain, similar to those of other common hepatobiliary diseases, so the majority of patients neglect early-mild symptoms and are diagnosed at an advanced stage, further increasing the difficulty of CCA treatment[9]. Therefore, more specific CCA biomarkers are urgently required. In addition, the targeted drug blocks the growth and spread of cancer cells in CCA treatment by targeting the specific genes, proteins, or tissue environment of the cancer that contributes to its growth and survival [10]. Therefore, the identification of new targeted drugs would be helpful for neoadjuvant and adjuvant therapy in CCA.

Bioinformatics is a field combining biology, medicine, mathematics, and computer science [11]. While omics, particularly genomics, have become increasingly popular in recent years, bioinformatics can help scientists easily find useful information among the large and complex amount of genome data [12]. Therefore, it is more reliable to analyze the underlying molecular pathway, genetic alternation, prognosis, potential target gene, and novel biomarkers in cancer through the utilization of bioinformatics for processing multi-omics biological data. Combined high-throughput technology and computer-aided analysis would be helpful for identifying new CCA markers and novel target molecules, which have huge clinic value for overcoming challenges during early diagnosis, proper treatment, and ideal prognosis, thereby decreasing CCA morbidity and mortality.

Virtual screening is a promising tool for drug discovery and development[13]. In comparison to random screening, virtual screening achieves a hundred to thousand-fold efficiency for drug hit rate enrichment [14, 15]. There are generally two different virtual screening strategies: docking-based virtual screening and pharmacophore-based virtual screening [16]. Researchers have found pharmacophore-based virtual screening to outperform docking-based virtual screening for the retrieval of actives from the databases, making pharmacophore-based virtual screening a powerful drug discovery method [17].

This study identifies and analyzes differentially expressed genes (DEGs) from The Cancer Genome Atlas (TCGA) and Gene Expression Omnibus (GEO) databases using the bioinformatics approach. The potential key hub genes related to CCA are identified through the construction of a protein-protein interaction (PPI) network. For tumor down-regulated hub genes, the regulatory miRNA are predicted and the content change is confirmed in several GEO databases and biliary cell lines. For tumor up-regulated hub genes, the expression level in cells is validated and some compounds that exert potential gene-targeted activities are screened using pharmacophore-based virtual screening. Molecular dynamics simulations are adopted as a means of confirming the degree of binding between the key target and drug candidate. Finally, in vitro experiments are utilized to provide further confirmation of the anti-tumor activity of the drug candidate in cholangiocarcinoma cells.

## Materials and Methods

### Data Collection

Two gene expression profiles (GSE26566 and GSE45001) and three miRNA expression profiles (GSE113740, GSE113486, and GSE112264) were retrieved from the GEO (https://www.ncbi.nlm.nih.gov/geo/) database [18]. GSE26566 contains 104 CCA tumor samples and 6 normal samples, while GSE45001 contains 10 CCA tumor samples and 10 normal samples. GSE113740, GSE113486, and GSE112264 contain 25, 40, and 50 CCA tumor samples and 10, 100, and 41 normal samples respectively. The RNA sequencing datasets of 33 CCA tumor samples and eight normal samples, including clinicopathological information, were downloaded from the TCGA (TCGA-CHOL, https://tcga-data.nci.nih.gov/) database [19].

### Identification of Differentially Expressed Genes and miRNA

GSE26566, GSE45001, and TCGA-CHOL data were processed using RStudio software (version 4.1.0, https://www.rstudio.com/). For GSE26566 and GSE45001, the limma package was used to screen differentially expressed genes (DEGs) between tumor samples and normal samples [20]. With the TCGA-CHOL data, the edgeR package was used to screen DEGs [21]. GEO2R online analysis tool (https://www.ncbi.nlm.nih.gov/geo/geo2r/) was used for evaluating differentially expressed miRNA between CCA tumors and normal tissue samples [22]. All genes or miRNA with |log2FC|≥1 and adjust.*p* < 0.05 were considered to be of statistical significance. Venn diagram analysis was used as a means of obtaining the overlapping DEGs in the GSE26566, GSE45001, and TCGA-CHOL data.

### GO and KEGG Enrichment Analysis

Database for Annotation, Visualization, and Integrated Discovery (DAVID, https://david.ncifcrf.gov/) online web server was used for performing Gene Ontology (GO) analysis and Kyoto Encyclopedia of Genes and Genomes (KEGG) pathway enrichment analysis [23]. Terms with *p* < 0.05 were considered to be significant. Analytic results in DAVID were plotted using the Hiplot (https://hiplot.com.cn/) data visualization webserver.

### Construction of Protein-protein Interaction Network and Analysis of Clusters

Protein-protein interactions (PPI) of the DEGs were constructed by using the STRING (https://string-db.org/) database [24]. A confidence score of ≥ 0.4 was considered to be significant. The results of the PPI network were then downloaded and embellished using Cytoscape (version 3.8.2) [25]. The plugin Molecular Complex Detection (MCODE) was utilized for screening key gene clusters with haircut on and degree cut-off, node score cut-off, k-core, max depth set as 2, 0.2, 2, 100.

### Hub Gene Analysis

CytoHubba, which is a Cytoscape plugin, was used for identifying the targets with higher degrees in different computation methods [26]. 10 Cytohubba algorithms, including BottleNeck, EPC, Betweenness, Closeness, Degree, MCC, MNC, Radiality, EcCentricity, and Stress were used for gene ranking. The common genes ranking in the top 10 for different algorithms were listed and those with an occurrence rate of ≥50% were considered to be hub genes. The expression of hub genes in 23 different cancers was analyzed by the GEPIA (http://gepia.cancer-pku.cn/) webserver based on information that was derived from the TCGA database [27].

### Prediction of miRNA Regulating Hub Gene Expression

The TargetScan (human7.2, http://www.targetscan.org/vert_72/) webserver was used for predicting hub genes-regulatory miRNA [28]. Only broadly conserved miRNA, which presents greater reliability and prediction accuracy, was recorded for further analysis.

### Survival Analysis

The GEPIA webserver was used for performing prognostic analysis of hub genes in CCA [27]. Patients were split using auto selection, and the hazard ratio with 95% confidence intervals and log-rank *p* values were then computed.

### Pharmacophore-Based Virtual Screening

Pharmacophore-based screening was performed using the online sever Pharmit (https://pharmit.csb.pitt.edu/)[30]. The pharmacophore models were constructed based on CDK1/Cks2 complex (PDB: 6GU7), which was co-crystallized with AZD5438 [31]. For the virtual screening program, most pre-installed Pharmit parameters were left unchanged. One hydrogen donor and five hydrogen acceptors were locked for compound screening in the Molport library. The top 10 hit compounds were listed based on their scores.

### Molecular Dynamics Simulation

The crystal structure of 6GU7 was derived from the Protein Data Bank. The basic structure of Molport-003-703-800 was derived from the Molport library and the geometry structure was further optimized using the Gaussian09 program with B3LYP/6-31G (d,p) basis sets. Molecular docking between 6GU7 and Molport-003-703-800 was performed by using AutoDock Vina software. The minimum energy conformation from 100 docking results was chosen for the subsequent molecular dynamics simulation.

The Gromacs2019.4 program was used to simulate the molecular dynamics of the selected docked poses. The force field parameters for small molecules were calculated using the online webserver LigParGen (http://zarbi.chem.yale.edu/ligpargen/). The entire system was solvated in TIP3P water molecules in a box with 10Å×10Å×10Å dimensions. Before the MD simulation commenced, 2,500 steps of energy minimization were performed using the steepest descent and conjugate gradient method. The constraints were subsequently released and the same 5,000 steps of energy minimization were run for the entire system, including 2,500 steepest descent method steps and 2,500 conjugate gradient method steps. During the molecular dynamics simulations, long-range electrostatic interactions were treated using the particle mesh Ewald (PME) method. The time step was set as 2 fs, and LINCS was applied as a means of constraining the bonds that connect hydrogen atoms. A nonbonded interaction cut-off of 10Å was utilized. Temperature and pressure were maintained at 300 K and 1 atm through use of the V-rescale temperature and Berendsen pressure coupling method. The system was finally submitted to 100 ns molecular dynamics simulations. Simulation results were visualized by using the Gromacs embedded program and PyMOL (https://pymol.org/2/). The g_mmpbsa program was used for calculating the binding free energy between protein and ligand. The Discovery Studio Visualizer 2020 was employed for the investigation of protein-ligand interactions.

### Reagents and Cell Culture

Molport-003-703-800 (M078) was purchased from Molport (https://www.molport.com/, Riga, Latvia). The human cholangiocarcinoma cell lines RBE cells and human intrahepatic biliary epithelial cells HiBEC were acquired from the Institute of Biochemistry and Cell Biology, Chinese Academy of Sciences (China). The cells were cultured in RPMI-1640 (Gibco, Germany) medium, which was supplemented with 10% fetal bovine serum (FBS) and 1× antibiotic/antimycotic (Gibco).

### Real-Time qPCR Assay

Total RNA was extracted from cells by using TRIzol reagent (Molecular Research Centre, USA). RNA was reverse transcribed by using the PrimeScript RT reagent Kit (Takara, Japan). Real-time qPCR was performed using TB Green Premix Ex Taq II (Takara), on a CFX96 Touch Real-Time PCR Detection System (Bio-Rad). Target gene sequences can be seen in Table S1. Target transcript levels were normalized to *Actb* levels.

### Cell Viability Assay

The RBE cell line was placed into 96-well plates at a density of 5,000 cells/well. Different concentrations of the test compounds were then added to the cells. Following 72 h of incubation, 20 μl MTT solution (Solarbio Life Science, China) was added to each well, and the cells were then incubated for a further 4 h. The medium was then removed, and 150 μl DMSO solution was added to each well. Cell viability was calculated by taking measurements of the OD490 using a SpectraMax M5 Multi-Mode Microplate Reader.

### Western Blot

Proteins were isolated using RIPA lysis and extraction buffer (catalog number: 89900; Thermo Fisher), and protein concentrations were measured with a Pierce BCA Protein Assay Kit. Protein lysates were separated through the use of sodium dodecyl sulfate-polyacrylamide gel electrophoresis before being transferred to polyvinylidene fluoride membranes. The membranes were blocked with skim milk for 1 h at room temperature, before being incubated overnight with primary antibodies at 4°C. Secondary antibodies were applied at room temperature for one hour. Immunoreactivity was visualized by using an enhanced chemiluminescence reagent (Bio-Rad, USA).

### Scratch-Wound Migration Assay

RBE cells were seeded in 6-well plates where they were allowed to grow until confluent. The confluent cells were wounded by being scraped with a sterile 0.2-ml-pipette tip. The culture medium was replaced with fresh medium containing 0, 2, 5, 10, 25 μM of Molport-003-703-800 and cultured for 48 h. The rate of wound closure was monitored by images that were captured using a phase-contrast microscope.

### Cell Apoptosis Analysis with Flow Cytometry

A FITC Annexin V Apoptosis Detection Kit (BD Biosciences, USA) was used to conduct apoptosis analysis. The RBE cells were treated with Molport-003-703-800 for 24 h in gradient concentration. The cells were then harvested before being resuspended in 1× binding buffer and stained with propidium iodide (PI) /FITC-Annexin V. Staining was terminated after 15 min with 400 μl of 1× binding buffer. The cells were finally analyzed using BD Accuri C6 Cytometer (BD Biosciences, USA).

### Tunel Staining Assay

RBE cells were permeabilized with 4% paraformaldehyde fixation for 10 min and 0.1% Triton X-100 for 10 min. They were then blocked with 5% bovine serum albumin for 30 min. 50 μl TUNEL detection solution was added to the sample and then incubated in the dark at 37°C for one hour. Images were obtained via a fluorescent microscope (200 × amplification; Nikon, Japan).

### Statistical Analysis

Kolmogorov–Smirnov test was used for validating the normal distribution of all data. Student’s t-test was used as a means of comparing two groups of data. One-way ANOVA and Dunnett’s post-hoc test were used for comparing more than two groups of data. All data was expressed as mean ± SEM. Prism 8.0 software (GraphPad, USA) was used for statistical analysis and statistical significance was set as *p* < 0.05.

## Results

### Identification of DEGs in Cholangiocarcinoma

**Fig. 1:**
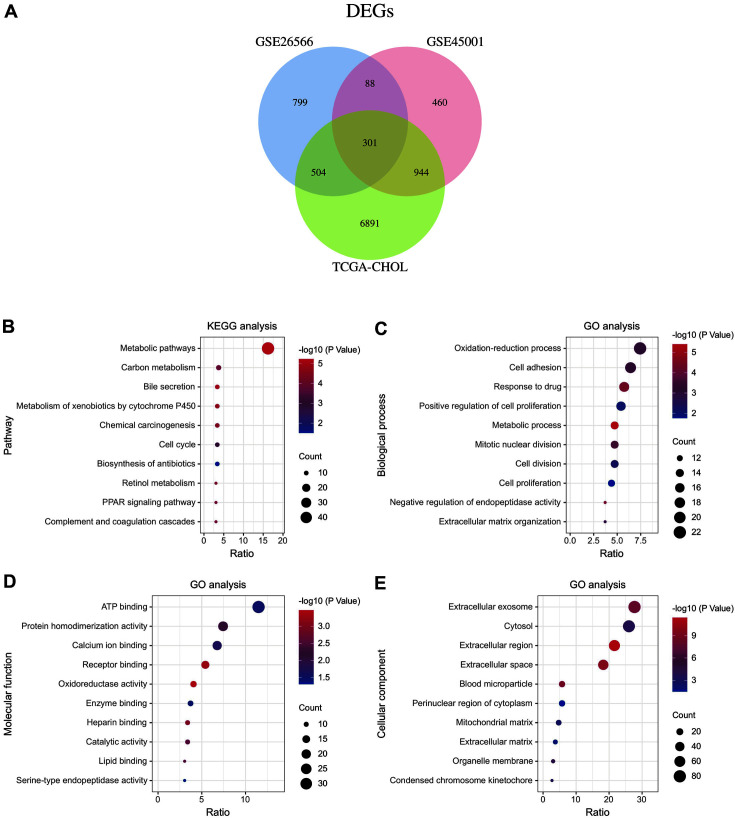
The total DEGs and their KEGG/GO analysis in CCA. (**A**) Venn diagram showed the overlapped DEGs in TCGA-CHOL, GSE26566, and GSE45001 databases. (**B**) The top 10 enriched KEGG pathways. (C-E) The top 10 enriched GO terms in biological process (**C**), molecular function (**D**), and cellular component (**E**).

DEGs in cholangiocarcinoma (CCA) were retrieved from both the TCGA and GEO databases. In the GEO database, the GSE26566 dataset contained 104 tumor cases and 6 normal cases, while GSE45001 contained 10 tumor cases and 10 normal cases. In the TCGA database, there were 33 tumor cases and 8 normal cases that were associated with CCA (TCGA-CHOL). Based on the parameters of an adjust.*p* value < 0.05 and |log2FC|≥1, 1,692 DEGs were identified in GSE26566, 1,793 DEGs were identified in GSE45001, and 8,640 DEGs were identified from TCGA data. In addition, Venn diagram analysis revealed there to be 301 common DEGs in the three different datasets (Fig. 1A). The overlapped DEGs were considered to be of great significance and were recorded for further analysis.

### Functional and Pathway Enrichment Analysis of the DEGs

GO and KEGG analyses were performed on the 301 overlapping DEGs. With KEGG pathway enrichment, the top 10 involved pathways were listed and it was noted that several pathways, including metabolism pathway, bile secretion, cell cycle, PPAR signaling pathway, and complement and coagulation cascades, were mainly affected by CCA (Fig. 1B). In addition, GO analysis revealed CCA-induced alternation in three terms. Regarding the biological process, the overlapped DEGs were significantly enriched in the oxidation-reduction process, cell adhesion, metabolic process, cell proliferation, and extracellular matrix organization (Fig. 1C). Regarding the molecular function, the overlapped DEGs were significantly enriched in ATP binding, calcium ion binding, receptor binding, enzyme binding, and lipid binding (Fig. 1D). Regarding the cellular component, the overlapped DEGs were significantly enriched in the extracellular environment and cytosol (Fig. 1E).

### Construction of PPI Network and Analysis of Clusters

**Fig. 2:**
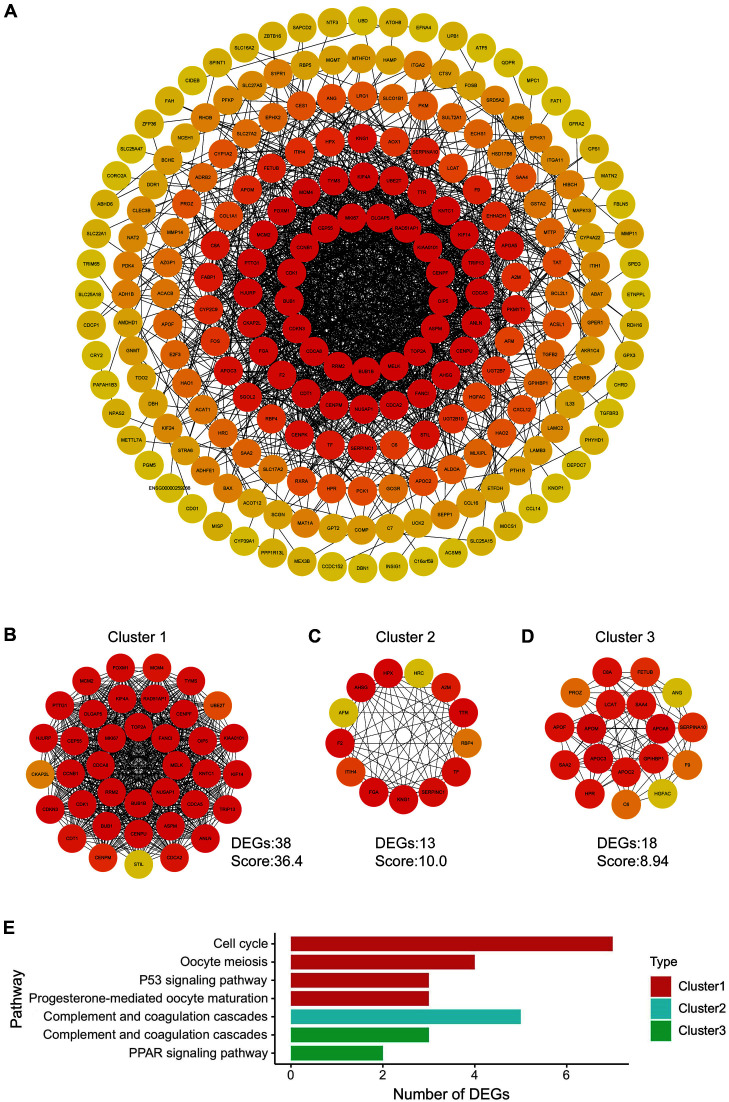
The PPI network construction of total DEGs. (**A**) The complete PPI network contains 219 nodes and 1421 edges. (B-D) The top 3 clusters seek out by the MCODE plugin. (**E**) The KEGG pathway enrichment of three clusters in panel B-D.

A PPI network is propitious to visualizing the relationship between the DEGs. Following analysis of the PPI by the STRING web server, the network was displayed using Cytoscape. PPI network interaction revealed 219 nodes and 1,421 edges on the map (Fig. 2A). Node color is proportional to the degree of connectivity with other targets. The MCODE plugin was then used for analyzing the entire PPI network and distinguishing between key gene clusters. The top three clusters were shown in the results based on their scores, and these clusters contained 38, 13, and 18 DEGs respectively (Figs. 2B-2D). In addition, KEGG analysis was performed on all 3 clusters. As the pathway enrichment results reveal, cluster 1 was significantly involved in cell proliferation, which is characteristic for almost all types of cancer, while clusters 2 and 3 were both found to have a strong correlation to complement and coagulation cascades (Fig. 2E). These results prove that CCA mainly results in the alternation of cell proliferation and complement and coagulation cascades.

### Hub Gene Identification

**Fig. 3:**
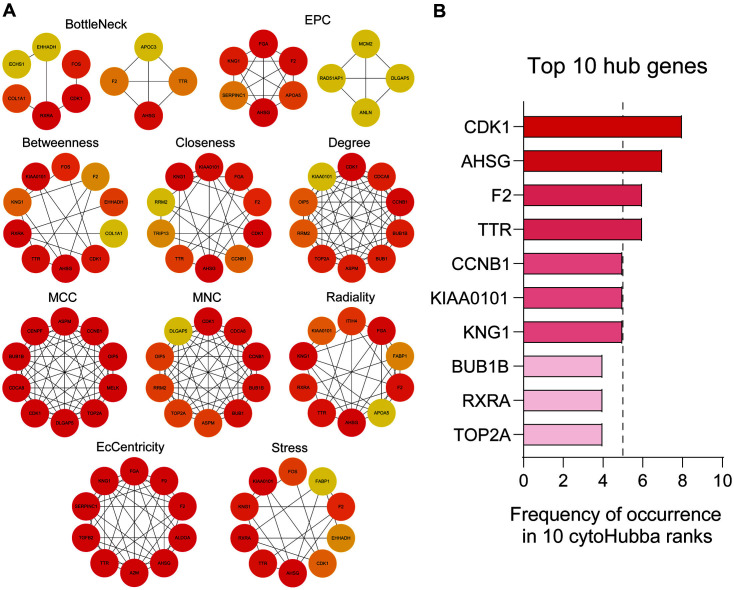
The hub gene identification. (**A**) The top 10 genes that ranked by the 10 algorithms in Cytohubba. (**B**) The top 10 genes that ranked by the occurrence rate in panel A.

Hub genes were chosen from the PPI network, in addition to using the cytoHubba following the construction of the PPI network. 10 different algorithms, including BottleNeck, EPC, Betweenness, Closeness, Degree, MCC, MNC, Radiality, EcCentricity, and Stress, were selected for gene ranking. The top 10 genes were constructed into a network for each algorithm (Fig. 3A). As tremendous differences were observed between the different algorithms, gene occurrence frequency was further calculated. The top 10 genes with high frequency were listed, and seven genes, CDK1, AHSG, F2, TTR, CCNB1, KIAA0101, and KNG1, with a frequency of ≥50% were considered to be hub genes in CCA (Fig. 3B). Interestingly, AHSG, F2, TTR, and KNG1 were all mapped in cluster 2, whereas CDK1, CCNB1, and KIAA0101 were all mapped in cluster 1. As clusters 1 and 2 were enriched in totally different pathways, the seven hub genes were then divided into two groups for further discussion.

### Expression of AHSG, F2, TTR, and KNG1 in CCA

**Fig. 4:**
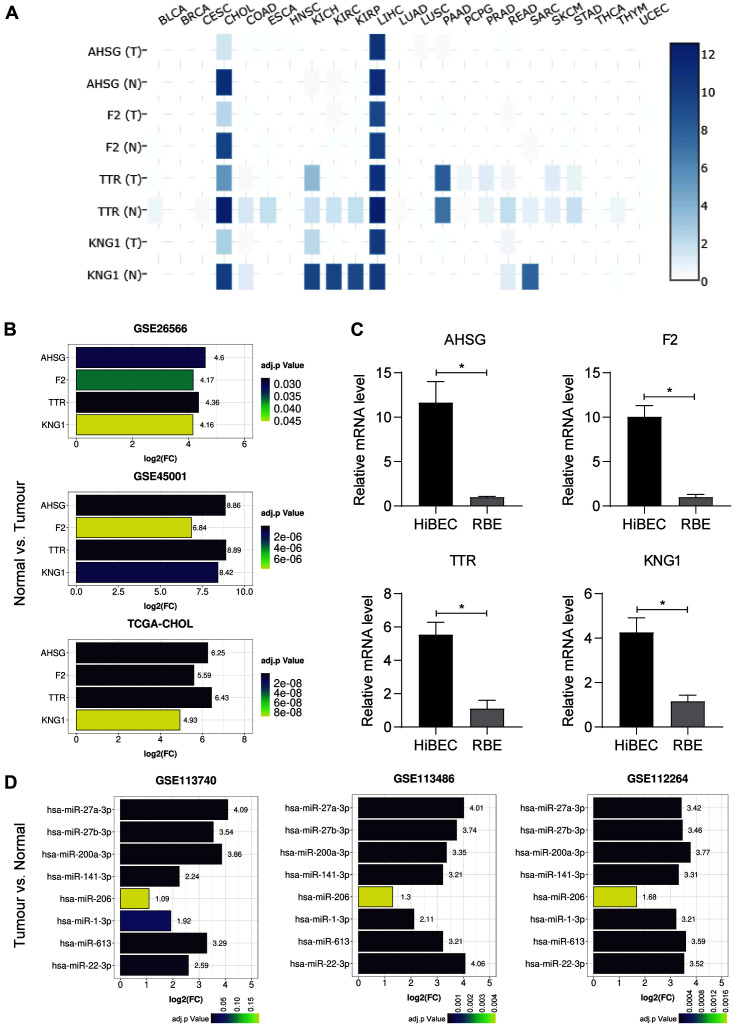
Verifying the mRNA/predicted miRNA expression of hub genes including AHSG, F2, TTR, and KNG1. (**A**) The heatmap of AHSG, F2, TTR, and KNG1 transcription in different cancer types and paired normal samples. T represents tumor tissues and N represents normal tissues. (**B**) The mRNA expression of AHSG, F2, TTR, and KNG1 in GSE26566, GSE45001, and TCGA-CHOL. (**C**) The mRNA expression differences of AHSG, F2, TTR, and KNG1 between RBE cells and HiBEC cells. (**D**) The miRNA expression in different GEO databases. Data are represented as mean ± SEM; *n* = 3 per group; *p < 0.05.

Firstly, the expression level of the four hub genes mapped in cluster 2 was analyzed through utilization of the GEPIA web service. Heat map results found AHSG, F2, TTR, and KNG1 all to be significantly downregulated in CCA (Fig. 4A). Furthermore, CCA also downregulated AHSG, F2, TTR, and KNG1 in GSE26566, GSE45001, and TCGA-CHOL datasets (Fig. 4B). These transcript differences between normal biliary cell line HiBEC and cholangiocarcinoma cell line RBE were also validated, and it was found that all four genes were silent in cholangiocarcinoma cells (Fig. 4C). These results indicate that AHSG, F2, TTR, and KNG1 could all be considered as novel CCA biomarkers. It should also be noted that TTR and KNG1 were also expressed and suppressed in other types of cancer, while AHSG and F2 were only expressed in CCA and liver hepatocellular carcinoma and only suppressed in CCA (Fig. 4A), which indicates that AHSG and F2 have higher specificity.

### miRNA Prediction and Expression Analysis

miRNA was considered the underlying mechanism that is responsible for AHSG, F2, TTR, and KNG1 downregulation in CCA. In order to verify this hypothesis, miRNA prediction was performed to confirm the potential regulatory miRNA of AHSG, F2, TTR, and KNG1 using the TargetScan web service. Eight miRNAs were found that broadly conserved with AHSG, F2, and TTR (Table 1). Conversely, no KNG1 conserved miRNA was found. In addition, the expression level of eight miRNAs was explored in three GEO datasets, GSE113740, GSE113486, and GSE112264. Results found all eight miRNAs to have increased significantly in the tumor sample of all three GEO datasets, while only hsa-miR-206 in GSE113740 presented an adjust.*p* value that was greater than 0.05 (Fig. 4D). The upregulated expression of these miRNA was found to be identical to the downregulated expression of AHSG, F2, and TTR, thereby proving the potential miRNA-dependent regulation of AHSG, F2, and TTR expressions in tumor samples.

### Expression and Prognosis of CDK1, CCNB1, and KIAA0101 in CCA

**Fig. 5:**
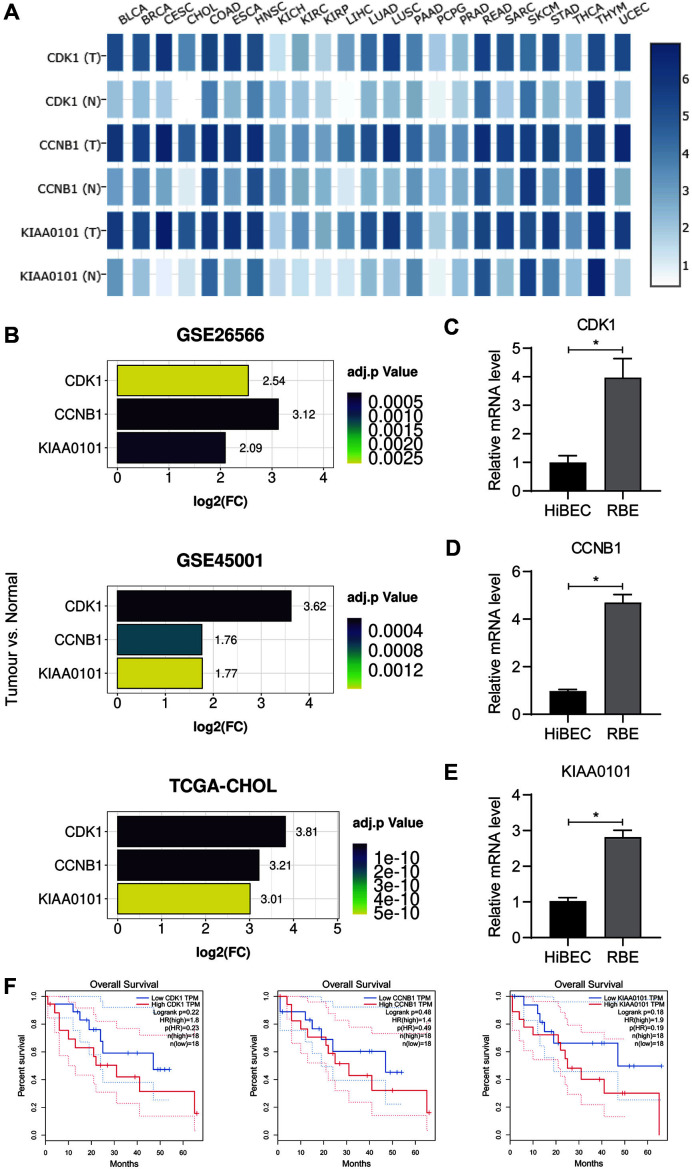
Verifying the mRNA expression and survival outcomes of hub genes including CDK1, CCNB1, and KIAA0101. (**A**) The heatmap of CDK1, CCNB1, and KIAA0101 transcription in different cancer types and paired normal samples. T represents tumor tissues and N represents normal tissues. (**B**) The mRNA expression of CDK1, CCNB1, and KIAA0101 in GSE26566, GSE45001, and TCGA-CHOL. (C-E) The mRNA expression differences of CDK1 (**C**), CCNB1 (**D**), and KIAA0101 (**E**) between RBE cells and HiBEC cells. (**F**) Correlation between the expression of CDK1, CCNB1, KIAA0101 and survival time in CCA. Data are represented as mean ± SEM; *n* = 3 per group; *p < 0.05.

Secondly, CDK1, CCNB1, and KIAA0101 were mapped in cluster 1 where they played a key role in cell cycle regulation. Heat map results from GEPIA found CDK1, CCNB1, and KIAA0101 all to be significantly upregulated in CCA (Fig. 5A). In addition, these upregulating trends were further confirmed in the GSE26566, GSE45001, and TCGA-CHOL datasets (Fig. 5B). Similarly, the mRNA levels of the three genes were found to be richer in RBE cells than HiBEC cells (Figs. 5C-5E). Prognostic analysis, which is supported by the GEPIA web service, found that lower expression levels of CDK1, CCNB1, and KIAA0101 have better overall survival in CCA, despite the p-value of >0.05 presenting no statistically significant differences (Fig. 5F). Considering the p-values were still approximately 0.2, a relatively lower value that indicates potential variability, it was believed that there could be a false negative caused by the small sample size (*n* = 36) for survival analysis. It should be noted that infinite proliferation potential is a common feature for most types of cancer, so CDK1, CCNB1, and KIAA0101 were broadly expressed and upregulated in a variety of cancers, making them unsuitable for use as CCA biomarkers but suitable for therapeutical targets.

### Potential Targeted Drug

**Fig. 6:**
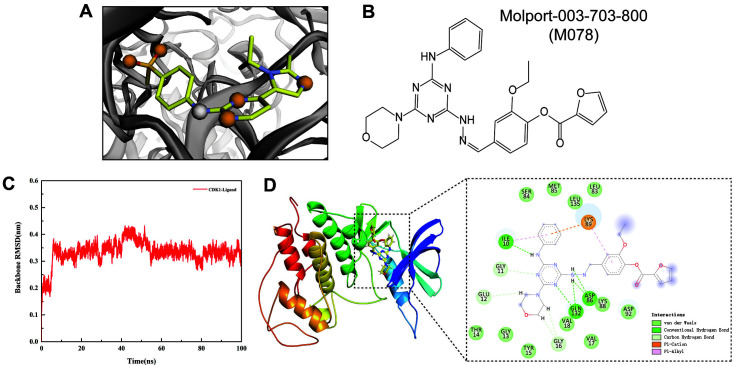
Pharmacophore-based virtual screening and molecular dynamics validation. (**A**) Pharmacophore models of PDB:6GU7. (**B**) The chemical structure of Molport-003-703-800 (M078). (**C**) The RMSD for 100ns molecular dynamics. (**D**) Docking results for Molport-003-703-800 with CDK1. The right square showed the detailed interaction between Molport-003- 703-800 with CDK1.

Currently, of CDK1, CCNB1, and KIAA0101, only CDK1 had been reported with the 3D crystal structure, thereby attracting attention from researchers and resulting in the subsequent development of several small molecular inhibitors. However, none of them were approved by FDA, so there was a requirement for more CDK1-targeted molecules. In order to achieve this, pharmacophores-based virtual screening was performed based on the crystal structure of CDK1/Cks2 in a complex with AZD5438 (PDB: 6GU7). In all pharmacophores, five hydrogen acceptors and one hydrogen donor were locked for searching in the Molport library (Fig. 6A). 480 hit compounds were found and the top 10 were listed (Table 2). The best hit compound was found to be Molport-003-703-800 (M078), and M078 structure was derived online (Fig. 6B).

In addition, molecular dynamics simulation was performed to provide further validation of the interactions between M078 and CDK1. In 100 ns molecular dynamics simulation, the root means standard deviation (RMSD) was calculated through a comparison with the initial position of complexes and the CDK1-M078 interaction displayed an RMSD of ~0.4 at 100 ns (Fig. 6C). It is notable that CDK1 had a small fluctuation before 10 ns and then had a tendency to stabilize, potentially due to interaction between protein and solvent water. For exploring binding affinity, the MM/PBSA approach was used to perform binding free energy calculations of the complexes (Table 3). The results found van der Waals interaction energy (ΔEvdW) to be a major interacting force between ligands and receptors that reached -42.47 kcal/mol. At the same time, electrostatic interaction (ΔEele) contributed -42.93 kcal/mol to the entire system. Generally, the total binding free energy (ΔGTot) reached -52.48 kcal/mol, representing the high affinity between M078 and CDK1. In addition, the 3D conformation was extracted from the final frame in molecular dynamics simulation (Fig. 6D). At the same time, protein-ligand interaction analysis that was performed by Discovery Studio Visualizer 2020 showed that Ile10, Asp86, and Gln132 of CDK1 formed hydrogen bonds with M078 to assist with protein-ligand binding and stabilize the entire complex (Fig. 6D). Therefore, M078 has the potential to be used as the CDK1-targeted drug.

### Validation of the Anti-tumor Activity of Molport-003-703-800 In Vitro

**Fig. 7:**
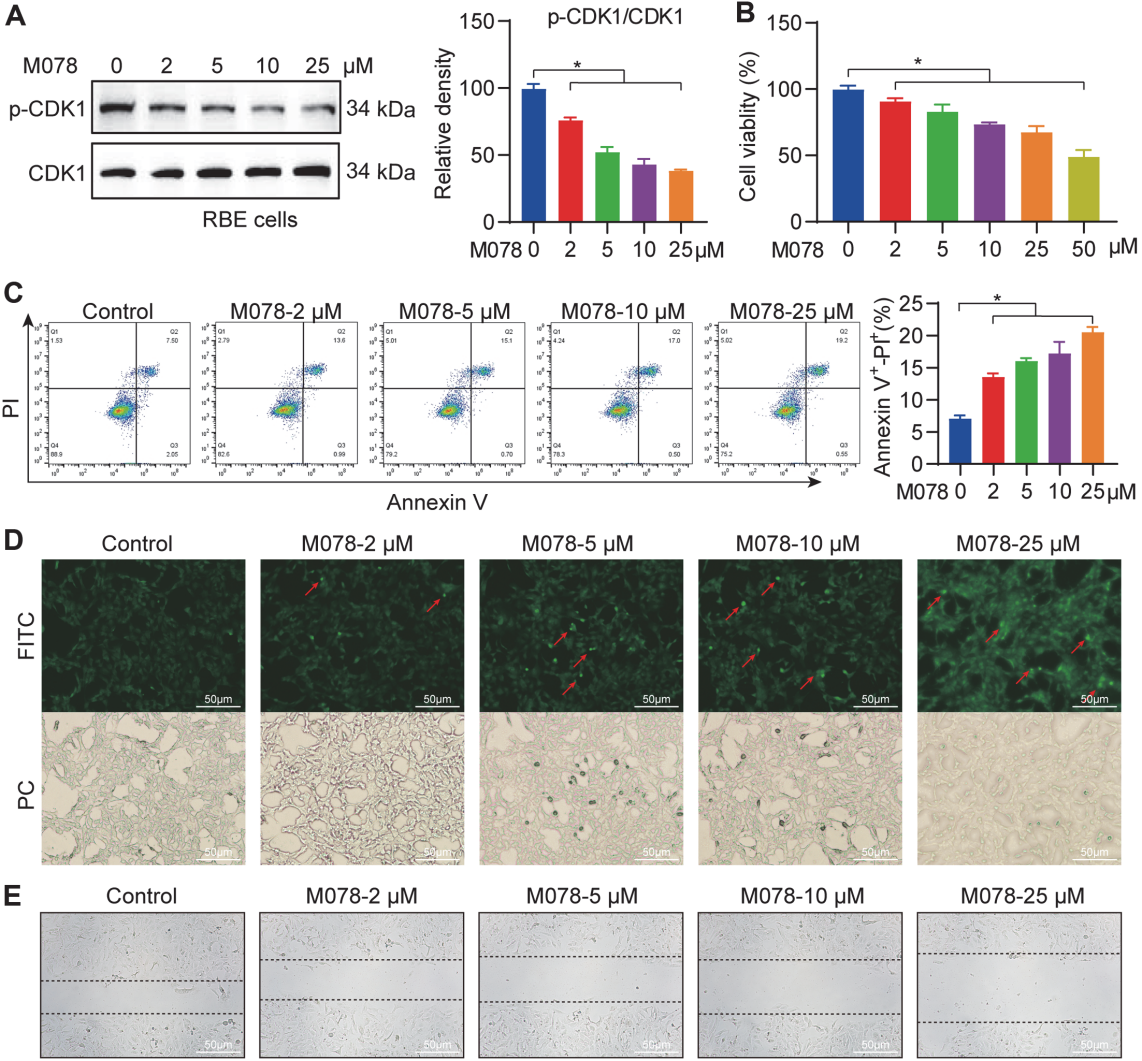
In vitro validation of the anti-tumor activity of Molport-003-703-800. (**A**) The protein level of CDK1 and phosphorylated CDK1 in RBE cells treated with 0, 2, 5, 10, 25 μM M078 for 1 h. The right panel showed the densitometric quantifications. (**B**) Cell viability of RBE cells treated with 0, 2, 5, 10, 25, 50 μM M078 for 72 h. (**C**) Flow cytometry analysis of RBE cells (treated with 0, 2, 5, 10, 25 μM M078 for 24 h) stained with PI/Annexin V. Q2 area (Annexin V^+^-PI^+^) represents the cell apoptosis. The right panel showed the quantification of Q2 area. (**D**) Tunel staining of RBE cells treated with 0, 2, 5, 10, 25 μM M078 for 12 h. The red arrow represents the positive focus. PC means phase contrast. (**E**) Scratch-wound migration image of RBE cells treated with 0, 2, 5, 10, 25 μM M078 for 48 h. Data are represented as mean ± SEM; *n* = 3 per group; *p < 0.05.

Following the identification of M078 as the potential targeted drug for CDK1, it was hypothesized that M078 could inhibit the activation of CDK1, thereby restraining the proliferation of cancer cells. As anticipated, in the in vitro study, the CDK1 phosphorylation, which is the active form of CDK1, was inhibited by M078 treatment of the RBE cells (Fig. 7A). In addition, M078 treatment lowered the viability of RBE cells in a dose-dependent manner (Fig. 7B). In addition, according to the Annexin V/PI staining assay and Tunel staining assay results, M078 treatment significantly promoted the apoptosis of RBE, which was indicated by an increase in Annexin V+-PI+ fractions and the Tunel positive area (Figs. 7C and 7D). The scratch-wound assay results also found M078 treatment to reduce RBE migration (Fig. 7E). These results indicate that M078 regulated the cholangiocarcinoma cell proliferation and apoptosis as a means of disrupting cell growth by blocking CDK1 activity.

## Discussion

In this study, differential RNA expression data for CCA patients was collected from the TCGA and GEO databases. Over 300 common DEGs were identified by Venn analysis. By using KEGG analysis, GO analysis, and PPI network visualization, seven hub genes (including AHSG, F2, TTR, KNG1, CDK1, CCNB1, and KIAA0101) mapped in two clusters were identified. AHSG, F2, TTR, and KNG1, are focused on the complement and coagulation cascades and play a different role in the regulation of coagulation response. AHSG, also known as fetuin-A, has been proven to present a negative correlation with coagulation activation in preeclampsia [32]. F2 encodes the thrombin, and this is a major component in coagulation cascades that converts fibrinogen into insoluble fibrin strands [33]. TTR and TTR amyloidosis can potentially activate the coagulation and fibrinolytic systems [34]. KNG1 has two different preferences in splicing, thereby generating either high molecular weight kininogen (HMWK) or low molecular weight kininogen (LMWK) [35]. HMWK is essential in blood coagulation and helps position optimally prekallikrein and factor XI next to factor XII [36]. Although these four genes have an opposite function in coagulation systems, they were all found to coincidently decrease in CCA in this study, further confusing the relationship between coagulation response and CCA. In addition, disseminated intravascular coagulopathy (DIC) is a common complication of solid tumors that is accompanied by the overproduction of coagulation factors [37]. However, there is a scarcity of clinical cases and the relationship between DIC and CCA cannot be verified. Despite the remaining confusion, AHSG, F2, TTR, and KNG1 all decreased in >16 folds with adj.*p* < 0.05 between normal with CCA patients in GSE26566, GSE45001, and TCGA-CHOL. AHSG and F2 are found to be predominantly expressed in the bile duct. These features prove the suitability of the four genes before being the novel markers for CCA, which may contribute to early CCA in a clinical setting.

microRNA is a small single-stranded non-coding RNA molecule that binds with mRNA by base-pairing with complementary sequences and cuts down the mRNA or downregulates the transcription activity of mRNA. Several miRNAs with the ability to target AHSG, F2, TTR, and KNG1 were predicted by seed region pairing. In addition, the high expression levels of the miRNAs in CCA were verified in GEO databases, which suggested that miRNA could be a possible mechanism responsible for CCA-induced AHSG, F2, TTR, and KNG1 silencing. It is notable that a recent bioinformatic study reported the DNA methylation of AHSG and F2 also increased in CCA patients [38], meaning that it may be another molecular mechanism for decreasing RNA levels of AHSG and F2 in cancer.

CDK1, CCNB1, and KIAA0101 are mapped in another cluster that is involved in the cell cycle. CDK1 is a serine/threonine kinase that forms a complex with its cyclin partners, including CCNB1, and phosphorylates the target substrates to cell cycle progression[39]. Proliferating cell nuclear antigen (PCNA) works as a DNA clamp for the promotion of cell replication, and KIAA0101 is vital for this process by direct binding with PCNA [40, 41]. Limitless replicative potential and self-sufficiency in growth signals are two of the typical hallmarks of cancer [42]. These features benefit from the overexpression of CDK1, CCNB1, and KIAA0101, which have all been found to positively control cell proliferation. Therefore, inhibiting CDK1, CCNB1, and KIAA0101 may be an effective means for controlling tumorous growth. Of these three genes, CDK1 has been researched the most and could be a more appropriate target for cancer treatment. Approximately 30 different CDK1 inhibitors have been identified, none of which have been approved by the FDA due to their side effects or direct toxicity [43]. Therefore, new CDK1 targeted compounds were identified by pharmacophore-based virtual screening (PBVS), and Molport-003-703-800 (M078) was ultimately identified in the Molport library. The high affinity between M078 and CDK1 was confirmed through molecular docking and dynamics simulation.

Finally, M078 anti-tumor activity has been validated in cholangiocarcinoma RBE cell lines. It should be noted that the FDA-approved drugs for CCA treatment are currently pemigatinib and infigratinib, which are kinase inhibitors for the fibroblast growth factor receptor (FGFR) family [44]. M078 is different to these two FGFR inhibitors and provides a novel CDK1-targeted strategy, making it a strong candidate drug for the treatment of CCA. The future in vivo validation of M078 will be of great interest.

In conclusion, by performing progressive bioinformatics analysis, it was found that complement and coagulation cascades-related genes (including AHSG, F2, TTR, and KNG1) and cell cycle-related genes (including CDK1, CCNB1, and KIAA0101) were conspicuous in the process of CCA. AHSG, F2, TTR, and KNG1 were all specifically downregulated in CCA patients under the regulation of miRNA network, which could be the novel and specific biomarkers for CCA. In addition, CDK1, CCNB1, and KIAA0101 were found to be substantially overexpressed in CCA, which can promote the proliferation of tumor cells. Molport-003-703-800 is a new targeted drug that demonstrated a potent affinity with CDK1 and anti-tumor activity in vitro, making it a potential candidate drug for CCA chemotherapy. The new biomarkers and CDK1-targeted drug will make a significant contribution to improving CCA diagnosis, Raw measurements are available in the Supplemental files. Supplementary files 1-3 were analyses of sequencing results from GSE26566, GSE45001, TCGA-CHOL, respectively.

## Supplemental Materials

Supplementary data for this paper are available on-line only at http://jmb.or.kr.

## Figures and Tables

**Table 1:** Highly conserved human miRNA list predicted by TargetScan web server.

Symbol	Consequence	Position	Site type	Predict miRNA
AHSG	ACUGUGA	293-299 of 3' UTR	7mer-m8	hsa-miR-27b-3p hsa-miR-27a-3p
TTR	CAGUGUU	78-84 of 3' UTR	7mer-m8	hsa-miR-200a-3p hsa-miR-141-3p
	ACAUUCC	129-135 of 3' UTR	7mer-m8	hsa-miR-206 hsa-miR-1-3p hsa-miR-613
F2	GGCAGCU	126-132 of 3' UTR	7mer-m8	hsa-miR-22-3p
KNG1	Not found			

**Table 2:** Top 10 CDK1-targeted compounds that identified by pharmacophores-based virtual screening.

Rank	CDK1/Cks2 (6GU7)	Score	RMSD
1	Molport-003-703-800	-8.61	4.358
2	Molport-002-942-764	-8.60	2.171
3	Molport-002-696-165	-8.60	6.768
4	Molport-007-552-096	-8.59	1.363
5	Molport-000-779-098	-8.57	3.074
6	Molport-023-277-074	-8.57	3.260
7	Molport-002-685-121	-8.57	4.044
8	Molport-007-951-239	-8.57	4.861
9	Molport-002-694-975	-8.56	4.649
10	Molport-016-638-322	-8.54	3.059

**Table 3:** The results of MM/PBSA free energy calculation (kcal/mol).

Energy Component	ΔE_vdw_	ΔE_ele_	ΔG_Tot_
Protein-ligand	-42.47	-42.93	-52.48
