# Supplementary material for: Identification and Validation of Novel Biomarkers and Potential Targeted Drugs in Cholangiocarcinoma: Bioinformatics, Virtual Screening, and Biological Evaluation
Source: J Microbiol Biotechnol. 2022 Sep 13;32(10):1262–74. doi: 10.4014/jmb.2207.07037 (PMC9668091; doi:10.4014/jmb.2207.07037)
Supplement: Supplementary file 1 [file jmb-32-10-1262-supple.zip › JMB22-07037 Supple files/JMB22-07037_SUP_0_1.docx]

**Supplementary Table S1.** Primers used for real-time qPCR assay.

| **Gene** | **Species** | **Forward sequence** | **Reverse sequence** |
| --- | --- | --- | --- |
| *Ahsg* | homo | TCCTTGGGGATACAAACACACC | TACCACGGAAAACTTGCCATC |
| *F2* | homo | CACGGCTACGGATGTGTTCTG | ACCCTCAGCACAGTTACCTTC |
| *Ttr* | homo | TGGGAGCCATTTGCCTCTG | AGCCGTGGTGGAATAGGAGTA |
| *Kng1* | homo | TGCTCCAGGCTGCTACTAAGT | GGCTTCAGTTATGCGGTACAA |
| *Cdk1* | homo | AAACTACAGGTCAAGTGGTAGCC | TCCTGCATAAGCACATCCTGA |
| *Ccnb1* | homo | AACTTTCGCCTGAGCCTATTTT | TTGGTCTGACTGCTTGCTCTT |
| *Kiaa0101* | homo | GTGCTTGGTTCTTCCACCTCTG | CCTTTTTGCCACTTGGGAGTTGG |
| *Actb* | homo | CATGTACGTTGCTATCCAGGC | CTCCTTAATGTCACGCACGAT |
